# Dairy foods and bone health throughout the lifespan: a critical appraisal of the evidence

**DOI:** 10.1017/S0007114518003859

**Published:** 2019-01-14

**Authors:** Sandra Iuliano, Tom R. Hill

**Affiliations:** 1 Department of Endocrinology, University of Melbourne/Austin Health, Heidelberg, VIC 3084, Australia; 2 Institute of Cellular Medicine and Human Nutrition Research Centre, Newcastle University, Newcastle-Upon-Tyne NE2 4HH, UK

**Keywords:** Bone, Critical reviews, Dairy food, Research design

## Abstract

The consumption of high-Ca, high-protein dairy foods (i.e. milk, cheese, yogurt) is advocated for bone health across the lifespan to reduce the risk of low-trauma fractures. However, to date, the anti-fracture efficacy of dairy food consumption has not been demonstrated in randomised controlled trials but inferred from cross-sectional and prospective studies. The anti-fracture efficacy of dairy food consumption is plausible, but testing this requires a robust study design to ensure outcomes are suitably answering this important public health question. The evidence of skeletal benefits of dairy food consumption is equivocal, not because it may not be efficacious but because the study design and execution are often inadequate. The key issues are compliance with dietary intervention, dropouts, sample sizes and most importantly lack of deficiency before intervention. Without careful appraisal of the design and execution of available studies, precarious interpretations of outcomes may be made from these poorly designed or executed studies, without consideration of how study design may be improved. Dairy food interventions in children are further hampered by heterogeneity in growth: in particular sex and maturity-related differences in the magnitude, timing, location and surface-specific site of bone accrual. Outcomes of studies combining children of different sexes and maturity status may be masked or exaggerated by these differences in growth, so inaccurate conclusions are drawn from results. Until these critical issues in study design are considered in future dairy food interventions, the anti-fracture efficacy of dairy food consumption may remain unknown and continue to be based on conjecture.

Dairy foods, namely, milk, cheese and yogurt, are the principal sources of dietary Ca in developed countries, with an adequate consumption synonymous with optimal bone health. Evidence from observational and prospective studies supports this notion and is highly suggestive of this association; however, the most convincing proof of efficacy comes from well-designed randomised controlled studies. Equally, the association between fracture risk reduction and the consumption of dairy foods is also acquired from observational, case–control and prospective studies; but in the case of anti-fracture efficacy of dairy food consumption, this evidence is lacking, as these trials have not been reported. The aim of this narrative review is to critique the available evidence on dairy food consumption and bone health over the lifespan, with an emphasis on the importance of study design and execution.

## Method of data acquisition

A literature search was conducted on PubMed database between February and December 2017. The following Medical Subject Headings terms were used to identify randomised controlled studies of dairy foods: ‘dairy’ or ‘milk’ or ‘cheese’ or ‘yoghurt’ or ‘dietary calcium’ or ‘calcium-rich food’ and bone; ‘bone’. The two searches were combined with the term ‘randomized’ or ‘randomised’, resulting in the identification of 2087 journal articles. Following the removal of duplicates, the exclusion criteria of (i) animal studies, (ii) non-randomised trial, (iii) not a dairy food-based intervention, (iv) non-English articles and (v) bone-related measures not being a primary outcome were applied. The remaining full-text articles were reviewed and a manual search of reference lists conducted to identify any additional articles. Of the fifty-five remaining articles, an additional exclusion criteria of ‘dairy products used intentionally as a vehicle for nutrient delivery (e.g. vitamin K)’ was applied. Articles reporting interim or follow-up outcomes from the same study were also removed. These articles are mentioned but not discussed in detail in the following critical appraisal of the literature.

### Growth, bone structure and dairy food interventions: trying to hit a moving target

The lower fracture rates observed in elderly men compared with women may, in part, have their origins in sex differences in bone morphology from early in life. Males are born with a larger skeleton compared with females, implicating the potential of genetic or intrauterine factors in producing sex differences^(^ ^1^ ^)^. Higher testosterone levels have been measured in cord blood of newborn males, with levels peaking between 1 and 3 months, then decreasing to prepubertal levels by 7 months, compared with females in whom testosterone levels decline soon after birth to resemble prepubertal levels by 1 month^(^ ^2^ ^,^ ^3^ ^)^.

In both sexes, growth in crown–heel length is rapid soon after birth, slows within the first year then re-accelerates after 12 months of age^(^ ^4^ ^)^. However, this early growth is region-specific. Appendicular growth velocity accelerates, while axial growth continues at a slower rate. Before puberty, longitudinal growth does not differ by sex, which may suggest a common effector for both sexes, such as insulin-like growth factor-1 (IGF-1)^(^ ^5^ ^)^. At puberty when sex hormone levels rise, appendicular growth velocity declines while axial growth velocity accelerates^(^ ^6^ ^)^. So while the patterns of longitudinal growth are similar between the sexes, puberty occurs approximately 2 years earlier in females than in males. Males have longer legs but differ less from females in trunk length because of their longer period of prepubertal (appendicular) growth.

Greater bone width favouring males has been observed as early as 3 months of age^(1)^. Prepubertal male twins have a 5 % larger mid-femur cross-sectional area than their female co-twin that confers a 12 % greater resistance to bending, despite no difference in body weight. By the end of maturity in boy–girl twins, bending strength at the mid-femur is 3-fold greater in males compared with females but males are also heavier^(^ ^7^ ^)^. At puberty, in males, the outer (periosteal) surface continues to widen, while expansion of the inner (endosteal) surface slows then ceases to widen, resulting in thickening of the cortex but further from the central axis of the bone. In females, the expansion of the outer bone surface slows then ceases, while apposition occurs on the inner (endosteal) surface, also resulting in thickening of the cortex, but in a narrower bone that is closer to the central axis. Androgens, growth hormones and IGF-1 stimulate periosteal expansion in males, while oestrogen inhibits expansion but stimulates endosteal apposition in females. The differences in linear and radial growth velocities by region, sex and age, and sex differences in age of maturity indicate that nutritional factors that may promote bone growth will have differing effects depending on the timing and duration of exposure.

### Dairy foods, calcium and bone growth in children and adolescents

Dairy foods are the main source of Ca during growth, with between one- and two-thirds of Ca obtained from dairy products^(^ ^8^ ^,^ ^9^ ^)^. Despite variations in dietary recommendations for Ca intake in children, an insufficient intake of Ca is likely in the absence of dairy food, unless fortified foods or supplements are consumed. Gao *et al.* ^(^ ^10^ ^)^ used data from over 2000 adolescents from the National Health and Nutrition Examination Survey study and modelled dietary intake aligned to the guidelines, with and without dairy foods, and observed that adequate Ca was not achievable with regular food without the inclusion of dairy food and was only possible if Ca-fortified foods were consumed. In the USA, for children aged 2–18 years, the average Ca intake is approximately 950 mg/d of which 32–51 % is obtained directly from milk and cheese, thus emphasising the role of dairy foods as the principal dietary source of Ca^(^ ^11^ ^)^.

A limited number of randomised studies investigated the effect of dairy food consumption on bone accrual during growth (*n* 12) (Table 1)^(^ ^12^ ^–^ ^23^ ^)^. Amongst these studies issues of varying maturity levels of participants, adequate baseline Ca intakes, poor compliance and high dropout cloud interpretation of results highlight the challenges of interventions involving children.

**Table 1:** Summary of study characteristics and the main strengths and limitations of randomised dairy food-based interventions across the lifespan

Reference	Study characteristics	Strengths	Limitations
Growth			
Zhu *et al.* (2008)^(^ ^12^ ^)^	*n* 345 boys and girls aged 10 years Milk (Ca)/milk (Ca+vitamin D)/placebo 2 years	Dietary Ca <700 mg/d	Pubertal status not reported Compliance not reported
Gibbons *et al.* (2004)^(^ ^13^ ^)^	*n* 154 boys and girls aged 8–10 years Milk powder/placebo 18 months	80 % compliance (in those who completed)	Baseline Ca >900 mg/d >50 % withdrew
Iuliano-Burns *et al.* (2006)^(^ ^14^ ^)^	*n* 99 boys and girls aged 5–11 years Fortified foods (milk minerals)/placebo 10 months	80 % compliance	Dietary Ca >700 mg/d Small number of prepubertal children remaining
Du *et al.* (2004)^(^ ^15^ ^)^	*n* 757 girls aged 10 years Milk (Ca)/milk (vitamin D)/placebo 2 years	Dietary Ca <700 mg/d 100 % compliance	Dietary intake of milk reduced Uneven distribution of maturity levels
Lau *et al.* (2004)^(^ ^16^ ^)^	*n* 324 boys and girls aged 10 years Milk powder: 80 g or 40 g/placebo 18 months	Dietary Ca <500 mg/d School-based compliance 100 %	Pubertal status not reported at follow-up Compliance only reported for one group
Chevalley *et al.* 2005^(^ ^17^ ^)^	*n* 235 prepubertal boys aged 6–9 years Milk minerals (in food)/placebo 12 months	Single maturity level at baseline 98 % compliance	Dietary Ca >700 mg/d Summed bone mineral density changes from all five sites
Cheng *et al.* (2005)^(^ ^18^ ^)^	Girls aged 10–12 years Ca+vitamin D/cheese/placebo 2 years	70 % compliance Dietary Ca <700 mg/d	Uneven distribution of pubertal status Approximately 1/3 not followed up
Bonjour *et al.* (1997)^(^ ^19^ ^)^	*n* 149 girls aged 6–9 years Milk minerals (in food)/placebo 48 weeks	Single maturity level at baseline Food-based approach	Dietary Ca >700 mg/d Pubertal status not reported at follow-up
Merrilees *et al.* (2000)^(^ ^20^ ^)^	*n* 105 girls aged 15–16 years Dairy foods/placebo 2 years	Choice of dairy foods	Dietary Ca >700 mg/d
Iuliano-Burns *et al.* (2003)^(^ ^21^ ^)^	*n* 66 children mean age 8·8 years Milk minerals (in food)/placebo 8·5 months	Dietary Ca intake <700 mg/d	Pubertal status not reported post Reduced sample size for Ca only group
Chan *et al.* (1995)^(^ ^22^ ^)^	*n* 48 girls aged 9–13 years Dairy foods/placebo 12 months	Choice of dairy foods	Dietary Ca intake >700 mg/d Pubertal status not reported post
Cadogan *et al.* (1997)^(^ ^23^ ^)^	*n* 80 girls aged 12 years Milk/placebo 18 months	Stratified by maturity level	Dietary Ca intake >700 mg/d
Adulthood			
Kruger *et al.* (2006)^(^ ^37^ ^)^	*n* 92 women aged 20–35 years Milk (Ca)/milk (Ca+nutrients)/placebo 16 weeks		Short-term study Contained multiple nutrients
Kruger *et al.* (2016)^(^ ^38^ ^)^	*n* 136 premenopausal women Milk/milk (Ca+nutrients) 12 weeks	Dietary Ca <500 mg/d	Short term (12 weeks) Milk contained other nutrients
Woo *et al.* (2007)^(^ ^39^ ^)^	*n* 441 women aged 20–35 years Milk powder/placebo 2 years	Dietary Ca intake <500 mg/d	Controls heavier and higher bone mineral density Compliance dropped to 45 % in year 2
Older age			
Kruger *et al.* (2016)^(^ ^38^ ^)^	*n* 121 postmenopausal women Milk/milk (Ca+nutrients) 12 weeks	Dietary Ca <500 mg/d	Short term (12 weeks) Compliance not reported
Daly *et al.* (2006)^(^ ^40^ ^)^	*n* 167 men >50 years of age Milk (Ca+D)/placebo 2 years	85 % compliance	Fortified with Ca and vitamin D Dietary Ca >800 mg/d
Moschonis *et al.* (2010)^(^ ^41^ ^)^	*n* 66 postmenopausal women Dairy foods/placebo 30 months	Use of food	Vitamin D added part way through study Total body scan divided into region
Kruger *et al.* (2010)^(^ ^42^ ^)^	*n* 120 postmenopausal women Milk powder/placebo 16 weeks	98 % compliance Dietary Ca <500 mg/d	Short-term study (16 weeks) Milk fortified with other nutrients
Kruger *et al.* (2012)^(^ ^43^ ^)^	*n* 63 postmenopausal women Milk powder/placebo 12 weeks	98 % compliance Dietary Ca <500 mg/d	Short-term study (12 weeks) Milk fortified with other nutrients
Chee *et al.* (2003)^(^ ^45^ ^)^	*n* 200 postmenopausal women Milk (Ca)/placebo 2 years	92 % compliance Dietary Ca <500 mg/d	Groups not matched for bone mineral density at baseline
Lau *et al.* (2002)^(^ ^46^ ^)^	Postmenopausal women Milk (Ca)/placebo 3 years	Dietary Ca <500 mg/d >90 % compliance	Control group had higher baseline bone mineral density
Bonjour *et al.* (2012)^(^ ^49^ ^)^	*n* 71 postmenopausal women Cheese (Ca+D)/placebo 6 weeks	Dietary Ca <600 mg/d Compliance rated as high (value not reported)	Short-term study (6 weeks)
Heaney *et al.* (1999)^(^ ^50^ ^)^	*n* 204 men and women >55 years Milk/placebo 12 weeks	Dietary dairy food <1·5 servings	Short-term study (12 weeks)
Heaney *et al.* (2002)^(^ ^51^ ^)^	*n* 29 postmenopausal women Yogurt/placebo <2 weeks	Dietary Ca <600 mg/d 99 % compliance	Short-term study (<2 weeks)
Bonjour *et al.* (2009)^(^ ^59^ ^)^	Thirty-seven institutionalised women, mean age 84 years Cheese (Ca+vitamin D) 4 weeks	95 % compliance	Short-term study (4 weeks)
Recker *et al.* 1985^(^ ^70^ ^)^	*n* 30 postmenopausal women Milk/placebo 2 years	2-year intervention	Small sample size
Bonjour *et al.* (2013)^(^ ^76^ ^)^	*n* 59 institutionalised women Yogurt (Ca+D)/yogurt 10 weeks	89 % compliance	Dietary Ca >700 mg/d Short term (10 weeks)
Iuliano-Burns *et al.* (2012)^(^ ^77^ ^)^	*n* 84 institutionalised older adults Fortified foods (protein, Ca, vitamin D)/placebo 12 months	Food-based approach Dietary Ca <600 mg/d	40 % attrition

Identifying bone accrual resulting from puberty from that due to dairy food supplementation is challenging. Peak bone mineral accrual during growth may be as high as 400 g/year in males and 300 g/year in females, significantly more than what may be achievable with 1 year of dairy food supplementation^(^ ^24^ ^)^. Of the twelve studies, eleven did not control for Tanner staging (as a measure of sexual maturity) or failed to report Tanner staging before or after intervention, making it difficult to quantify the skeletal gains due to dairy food supplementation. For example, in a 24-month randomised study involving 195, 10–12-year-old girls, Cheng *et al.* ^(^ ^18^ ^)^ observed greater thickening of the cortex at the mid-tibia in girls who were >50 % compliant when assigned to cheese (providing approximately 1000 mg Ca) compared with Ca+vitamin D, Ca alone or placebo, but no group differences were observed for cross-sectional area of the tibia, suggesting bone accrual on the inner surface; a characteristic of puberty. At baseline, groups were not matched for pubertal status, and Tanner staging not reported at study end. When growth velocity was accounted for, treatment effects were no longer evident. The authors acknowledged the diversity of growth velocities likely masks the effect of supplementation as children transition through puberty.

Studies involving similarly aged males and females further exacerbate the potential for puberty to mask any benefits of dairy food supplementation. While sex differences in growth before puberty are negligible, females commence puberty about 2 years earlier than males do, so the earlier age of onset is accompanied by maturity-related acceleration in bone accrual. Iuliano-Burns *et al.* ^(^ ^14^ ^)^ randomised ninety-nine prepubertal children aged 5–11 years to 10 months of supplementation of 800 mg Ca daily from milk minerals, calcium carbonate or placebo, with a 12-month follow-up after supplementation. The greater gains in bone mineral content (BMC) at the pelvis in both treatment groups were not sustained after adjustment for multiple comparisons. However, when data were confined to those who remained prepubertal for the duration of the project, children assigned to the two treatment groups (milk minerals: *n* 25, calcium carbonate: *n* 30) accrued 8 % more BMC at the pelvis than controls (*n* 19), with the benefits still evident in the milk mineral group 12 months after discontinuation^(^ ^14^ ^)^. Changes to Ca absorption during maturation may further confound outcomes^(^ ^25^ ^,^ ^26^ ^)^.

Skeletal benefits of dairy food supplementation are unlikely if baseline Ca is sufficient. Of the twelve studies, seven included children with Ca intakes >700 mg/d, so depending on age (maturity status), participants were likely Ca replete^(^ ^13^ ^,^ ^14^ ^,^ ^17^ ^,^ ^19^ ^,^ ^20^ ^,^ ^22^ ^,^ ^23^ ^)^. This issue was highlighted in a meta-analysis conducted by Huncharek *et al.* ^(^ ^27^ ^)^. When all Ca-/dairy food-based intervention studies were pooled (*n* 12 studies, with 2460 participants), no differences were observed in total body BMC accrual between treated children and controls (2 g; 95 % CI –3, 7 g)^(^ ^27^ ^)^. However, baseline Ca intakes were low in three studies, which when analysed separately, a 50 g; 95 % CI 24, 77 g difference in total body BMC accrual between treatment and controls was observed. Two studies used milk fortified with Ca (approximately 600 mg) and one used fortified milk powder (approximately 840 mg Ca)^(^ ^27^ ^)^. Protein intake was also augmented (10–30 g per supplement).

Efficacy of treatment is compromised with poor compliance^(^ ^28^ ^)^. Of the twelve studies, seven reported compliance with dairy food-based interventions ranging from 60 to 100 %^(^ ^13^ ^–^ ^19^ ^)^. However, Gibbons *et al.* ^(^ ^13^ ^)^ reported 80 % compliance in those that remained in the study, but over 50 % of the study cohort withdrew. Lau *et al.* ^(^ ^16^ ^)^ observed better compliance when supplemental dairy foods were administered at school (100 %) compared with at home (58 %), and, despite 100 % compliance with supplemental milk (330 ml/d), Du *et al.* ^(^ ^15^ ^)^ observed that habitual milk intake declined during the intervention period, resulting in milk intake of approximately 250 ml daily during intervention, with no change observed in protein intake. Three studies reported Ca intake following supplementation, and all observed that habitual Ca intake and eating patterns reverted to pre-supplemented levels once intervention ceased^(^ ^12^ ^,^ ^13^ ^,^ ^20^ ^)^. Two issues highlighted by these inconsistencies in compliance are sustainability of the dairy food intake following intervention and the ability to quantify the amount of dairy food required for a skeletal benefit to be observed.

The process of randomisation is to disperse traits (e.g. sex, maturity) equally between groups and to reduce their potential to bias results. Large dropout numbers, or uneven withdrawal from allocated groups, violate this randomisation process and may also compromise the power to detect changes with treatment. For example, Gibbons *et al.* ^(^ ^13^ ^)^ reported a drop out rate of 53 % during an 18-month dairy food-based intervention and reported no clinical benefits with dairy food supplementation. However, it is difficult to conclude whether the intervention was effective, as the baseline Ca intake and maturity status of the remaining participants were not reported, and whether the remaining number of participants provided sufficient power to detect differences if present.

The skeletal benefits of dairy foods may relate to the suppression of bone remodelling with Ca, and/or the anabolic effect of stimulation of IGF-1 by protein or other synergistic benefits related to other constituents contained in dairy foods. The residual benefits observed by some authors would suggest an anabolic effect, while the reversal of benefits after cessation of dairy food supplementation suggests non-permanent changes perhaps due to suppression of bone remodelling in response to the elevated Ca intake, with remodelling returning to its previously higher rate once Ca intake is again lowered. Cadogan *et al.* ^(^ ^23^ ^)^ in their previously mentioned 18-month dairy food-based intervention involving 12-year-old girls observed an increase in IGF-1 over time with Ca-fortified milk consumption (*P*=0·023), but no change in bone markers. In a 7-d trial involving 8-year-old boys that compared the daily consumption of 1·5 litres of skimmed milk (*n* 12) to the equivalent protein intake from low-fat meat (*n* 12), a 19 % increase in IGF-1 and an 8 % increase in insulin-like growth factor-binding protein 3 were observed with enhanced milk but not with meat intake^(^ ^29^ ^)^. The authors suggested the milk may have a direct effect on hepatic production of IGF-1 or via stimulation of growth hormone. However, total energy intake increased in the milk (10 %), but not in the meat group, and the milk group gained weight. Re-feeding of protein–energy malnourished children also increases IGF-1, making it difficult to separate the effect of the increase in total energy compared with dairy food protein alone. The authors undertook a subsequent 7-d intervention involving 8-year-old boys that compared the consumption of 1·5 litres/d of high-casein skimmed milk with a high-whey skimmed milk and observed a 15 % increase in IGF-1 with casein but not with whey consumption^(^ ^30^ ^)^. The authors acknowledged that the casein content of milk is greater than whey content, so the effect may have been due to a higher protein intake.

### Dairy food intervention study design

The features of study design and execution that improves study quality include controlling for maturity, participants having low habitual dairy food intake, good compliance with intervention and sufficient study participants with limited dropout. Cadogan *et al.* ^(^ ^23^ ^)^ was the only reported study to stratify participants by Tanner staging (controlling for maturity at onset) before randomisation and observed gains in total body BMC and higher IGF-1 levels with the consumption of the additional dairy foods. No group differences in sex hormone levels were observed, suggesting that the groups were matched for maturity. To separate the region-specific skeletal benefits of dairy foods before, during or after puberty, it would require sufficient numbers of participants at baseline, to enable treatment effects to be investigated within each level of maturity (pre-, peri- or post-puberty). A skeletal response is more likely in those with low habitual dairy food (Ca) intake, so efficacy is more likely demonstrated when participants are selected on this basis. Division of participants into high and low consumers after randomisation, may violate the randomisation process or reduce the power to detect benefits of dairy food supplementation if present. Compliance is highest when supplementation was administered at school, so may be a suitable avenue of administration^(^ ^16^ ^)^. The provision of a variety of products/tastes may assist with compliance as ‘flavour fatigue’ reduces intake over time. Ongoing monitoring of supplemental and habitual food intake is beneficial, as reductions in normal dietary dairy food intake in response to supplementation or erosion of compliance over time may reduce the potential to observe skeletal changes as the magnitude of the difference in dairy food intake between intervention and controls is compromised. Furthermore, standardising serving sizes and methods of recording dietary intakes would facilitate comparisons between studies.

### Bone maintenance in adulthood and loss in later life: can dairy foods make a difference?

After puberty and leading into young adulthood, the bone continues to consolidate, albeit more slowly. Peak bone mass (PBM), which is defined as the amount of bone present at the end of skeletal maturation, is a major predictor of fracture risk in later life^(^ ^31^ ^)^. The attainment of PBM is thought to be achieved by approximately 30 years of age in both men and women, though PBM has been reported to be reached as early as the late teenage years or as late as the mid-30s depending on the skeletal site^(^ ^32^ ^)^.

Following the acquisition of PBM, bone continues to be remodelled, to remove and replace old and damaged bone. This process occurs at discrete foci on the bone surface. If within each focal point or basic multi-cellular unit (BMU) the amount of bone removed (resorption) and replaced (formation) is equal, then bone is neither lost nor gained. Bone loss is considered to commence some time during young adulthood due to a reduction in bone formation relative to bone resorption within each basic multicellular unit, so each BMU is effectively in a negative balance. Therefore, the number of BMU active at any point in time or the rate of remodelling would be a principal determinant of the rate of bone loss. Menopause in women is associated with increased remodelling intensity, or the number of remodelling sites initiated, due to the loss of oestrogen, which is marked by a rapid decrease in bone mineral density (BMD)^(^ ^34^ ^)^. Bone loss slows 3–5 years after menopause as a new steady state, but at a higher rate of remodelling, is achieved. Bone loss appears to be more gradual in men^(^ ^33^ ^,^ ^34^ ^)^.

A principal driver of apparent changes in bone mass is the intensity of remodelling or the number of discrete remodelling sites initiated. Ca is considered a moderate suppressor of remodelling intensity, so supplementation reduces the number of remodelling sites initiated, resulting in an apparent increase in BMD. Alternatively Ca deficiency is associated with increased bone remodelling intensity and results in an apparent reduction in BMD^(^ ^34^ ^)^. This is evident in Ca supplementation trials where markers of bone turnover are lowered with supplementation, but a reversal of skeletal benefits observed when Ca (and vitamin D) supplementation is ceased, likely resulting from remodelling intensity returning to pre-supplemented levels^(^ ^35^ ^)^.

During young adulthood, if the rate of remodelling is low, then further rate suppression of remodelling would be relatively small and likely difficult to detect. Therefore, detection of skeletal changes in dairy food (or Ca) supplementation trials in Ca-replete adults in whom remodelling rates are already low would likely be limited. During menopause when remodelling intensity is accelerated due to the rapid decline in oestrogen, the modest suppression of remodelling due to enhanced dairy food (or Ca) intake may also be challenging to detect. This was evident in the study by Dawson-Hughes & Dallal^(^ ^36^ ^)^ who observed maintenance of BMD at the lumbar spine, femoral neck and radius with 2 years of Ca supplementation (500 mg/d) in women who consumed <400 mg/d of Ca, who were >6 years post-menopause, but not in women within 5 years after menopause. Most reported dairy food interventions include women at least 5 years post-menopause, with a fewer number of interventions involving premenopausal women and a limited number involving older men (Table 1)^(^ ^37^ ^–^ ^40^ ^)^.

### Research design and skeletal effects of dairy food consumption

Dairy food interventions involving adults require the same stringent research design as those involving children; sufficient sample size to detect changes if present, low baseline dairy food (Ca) intake in participants, good compliance with intervention and limited dropouts to maintain the power to detect differences due to intervention. Some, but not all studies, fulfil these criteria. For example, despite including over 400 women with mean Ca intake of <500 mg/d in their 2-year dairy food-based intervention, Woo *et al.* ^(^ ^39^ ^)^ observed no group differences in total body, spine or hip BMD using intention-to-treat analysis; compliance fell from 75 % in the first year to 45 % in the second year. A <1 % difference in spine BMD at 6 months, but no other time point, was observed using per protocol analysis, which may reflect a transient suppression of remodelling^(^ ^39^ ^)^. However, this cannot be confirmed as the reported serum C-terminal telopeptide of type 1 collagen (CTX) (bone resorption) changes were for all participants, not just those included in the per protocol analysis. In those studies that fulfil the design criteria, the majority have supplemented using dairy foods fortified with Ca, vitamin D and/or other nutrients^(^ ^37^ ^–^ ^47^ ^)^. While some of these nutrients are inherently found in dairy foods, the amounts are greater than naturally occurring; therefore, difficulties arise when discerning the contribution of naturally occurring compared with added nutrients to skeletal changes observed. In some cases, dairy foods are used as a vehicle to deliver non-dairy product-related nutrients^(^ ^48^ ^)^. Furthermore, while the vitamin D content of milk is relatively limited (<0·05 μg/100 ml), vitamin D fortification is routine in some countries such as the USA and those in Scandinavia; therefore, the potential role of vitamin D in dairy foods needs to be considered especially when comparing studies from countries with and without routine fortification of dairy foods. The amount of vitamin D fortification and the size of a standard dairy food serving also vary between countries (and studies), which may potentially influence the magnitude of change in skeletal outcomes.

**Fig. 1:**
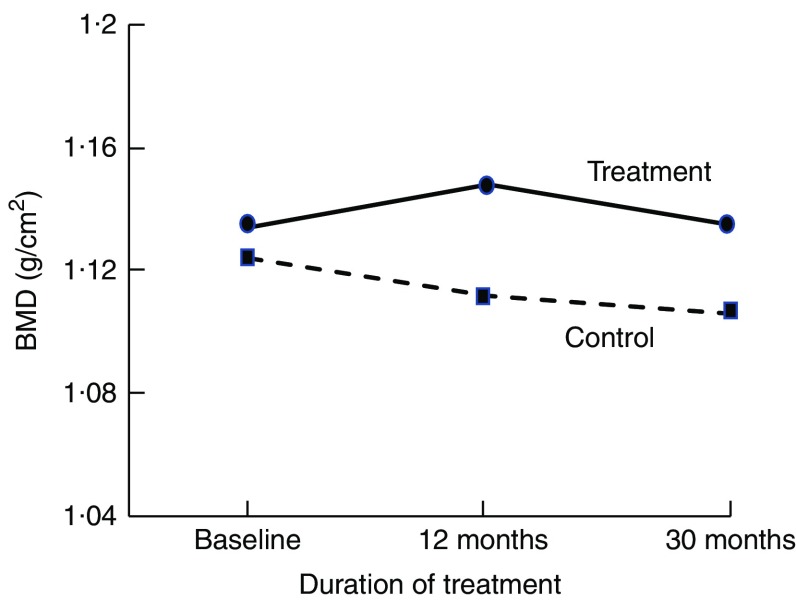
Changes in total body bone mineral density (BMD) at 12 and 20 months in postmenopausal women supplemented with fortified dairy foods compared with non-supplemented controls. Data are adapted from Moschonis *et al.* (2010)^(^ ^41^ ^)^.

Short-term (1–16 weeks) dairy food-based interventions in adults consuming <700 mg/d of Ca have demonstrated reductions in bone resorption (serum CTX and urinary *N*-telopeptide) of between 6 and 40 % within weeks of supplementation (Table 1)^(^ ^37^ ^,^ ^38^ ^,^ ^49^ ^)^. Only two short-term studies used non-fortified dairy foods^(^ ^50^ ^,^ ^51^ ^)^. Longer-term (1–3 years) dairy food-based interventions that have reported changes in BMD as an outcome support the notion of suppression of remodelling with dairy food supplementation. Most studies reported significant differences in the rates of change to BMD at the total body, lumbar spine and femoral sites between controls and intervention by 12 months; however, the magnitude of the differences was maintained for the subsequent period of observation (1–2 years)^(^ ^40^ ^,^ ^41^ ^,^ ^44^ ^,^ ^46^ ^)^. The rates of change in BMD in the subsequent years were similar between intervention and controls; the slopes of change are parallel (Fig. 1)^(^ ^41^ ^)^. Although fracture outcomes are not reported, Johansson *et al.* ^(^ ^52^ ^)^ in their meta-analysis of reference markers of bone turnover for prediction of fractures estimated that each standard deviation increase in CTX was associated with an 18 % increased risk of fracture (hazard ratio: 1·18, 95 % CI 1·08, 1·29) and a 23 % increased risk of hip fracture (hazard ratio: 1·23, 95 % CI 1·04, 1·47). Therefore, fracture risk reduction with dairy food supplementation is plausible.

Dairy foods may potentially have anabolic qualities due to other constituents in dairy foods, such as protein, or Ca in combination with these components. Protein deficiency is associated with accelerated bone resorption and impaired bone formation, and protein supplementation in older adults increases IGF-1 and promotes Ca absorption, potentially slowing bone loss^(^ ^53^ ^–^ ^55^ ^)^. Therefore, in the presence of protein insufficiency, improvements in serum IGF-1 levels is likely with dairy food supplementation. Defining protein requirement in older adults is challenging, but intake of 1·0–1·2 g/kg body weight are considered appropriate for healthy older adults, with levels of between 1·2 and 1·5 g/kg body weight suggested for those with chronic or acute illnesses^(^ ^56^ ^)^.

Improvements in IGF-1 levels have been observed in older adults when supplemented with dairy foods. Manios *et al.* ^(^ ^57^ ^)^ observed 13 % higher IGF-1 levels at 5 months in dairy food supplemented compared with non-supplemented postmenopausal women. Baseline protein intake was 0·7 g/kg body weight, and protein intake was augmented by approximately 12 g. No group differences in IGF-1 were observed at 12 months^(^ ^57^ ^)^. Kerstetter *et al.* ^(^ ^58^ ^)^ used a whey protein supplement in elderly men and women and observed approximately 6 % higher IGF-1 levels in supplemented than in controls at 18 months but not at 9 months. Baseline protein intake was approximately 1·0 g/kg body weight and protein intake increased by approximately 20 g. Bonjour *et al.* ^(^ ^59^ ^)^ observed a 17 % increase in IGF-1 in elderly institutionalised women after 1 month of supplementation using fortified soft cheese providing approximately 11 g of protein daily.

**Fig. 2:**
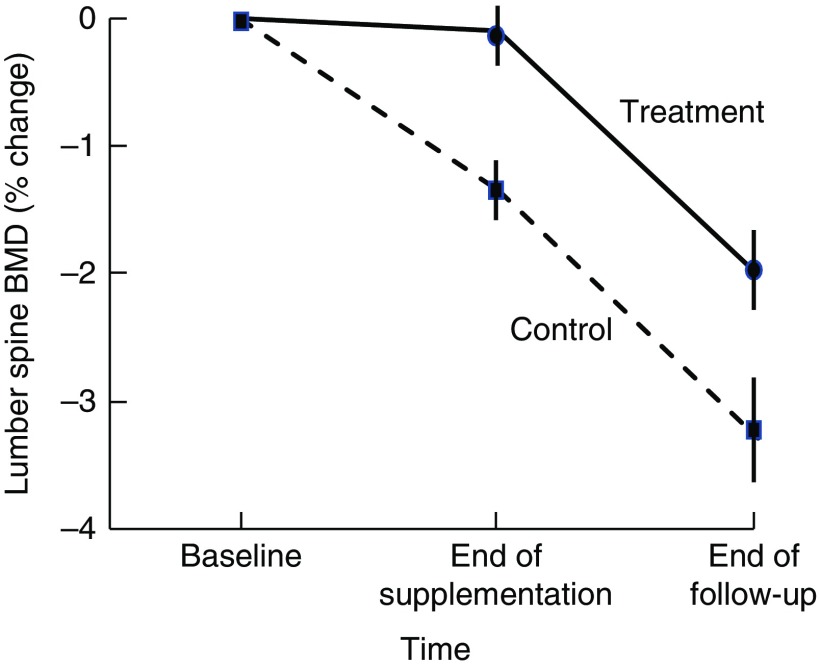
Changes in lumbar spine bone mineral density (BMD) in postmenopausal women supplemented with fortified milk powder or not receiving supplementation for 24 and 21 months following the discontinuation of treatment. Data are adapted from Ting *et al.* (2007)^(^ ^44^ ^)^.

Higher BMD has been observed in intervention than in control participants 18–21 months after cessation of dairy food supplementation, which is suggestive of residual skeletal benefits of dairy food supplementation^(^ ^44^ ^,^ ^60^ ^)^. Daly *et al.* ^(^ ^60^ ^)^ observed that 18 months after cessation of 2 years of dairy food supplementation in elderly males, the rate of change in BMD at the femoral neck favoured supplementation by 1·4 % (95 % CI 0·1, 2·7, *P*<0·05). Ting *et al.* ^(^ ^44^ ^)^ followed up postmenopausal female participants 21 months after cessation of 2 years of dairy food supplementation; and relative to baseline, the reduction in BMD observed at 45 months was less in the supplemented group than in controls at the lumbar spine (–2·01(SE 0·62) *v.* −3·29(SE 0·73)%) and femoral neck (0·44(SE 0·58) *v.* –1·49(SE 0·56) %) (both *P*<0·05). However, Daly *et al.* did not observe group differences in the rate of change in femoral neck BMD during the non-supplementation period, and Ting *et al.* ^(^ ^44^ ^)^ did not report the rate of change in BMD during the non-supplemented period but when plotted, rates of change to BMD were similar during this phase in the supplemented and controls (Fig. 2). Dietary Ca intake remained high during the non-supplemented period in the previously supplemented females (768(SD 424) *v.* 474(SD193) mg/d, *P*<0·005).

### Dairy foods and fracture risk reduction: seeking the elusive Holy Grail

Fractures are a public health problem that increase morbidity and healthcare costs^(^ ^61^ ^)^. By 2050, the number of people aged over 60 years globally will more than double, growing to over two billion, and constituting 25 % of the population^(^ ^62^ ^)^. While longevity is increasing, morbidity remains unchanged, so the increasing proportion of the population that is elderly will disproportionately increase the burden imposed by morbidity and cost associated with fractures^(^ ^63^ ^)^. Hip fractures are the most costly, with the number of hip fractures estimate to be between 4·5 and 6·3 million by 2050^(^ ^64^ ^,^ ^65^ ^)^. Drug therapy for all older adults is not feasible, so methods are needed that are efficacious, safe, widely available and inexpensive. This public health approach aims to reduce fracture risk modestly but in the entire elderly population^(^ ^66^ ^)^. Low intake of Ca and protein increase fracture risk, are common in the elderly and are amenable to treatment^(^ ^67^ ^,^ ^68^ ^)^. Increasing dairy food (i.e. milk, cheese, yogurt) consumption to recommended levels can effectively remedy protein and Ca deficiencies and fulfil the requirement of a safe, widely available and inexpensive means of intervention.

What is missing is the demonstration of anti-fracture efficacy, not because it is not plausible that adequate dairy food consumption may reduce fracture risk, but because to date no randomised controlled, prospective study investigating the anti-fracture efficacy of dairy foods have been undertaken. The demonstration of anti-fracture efficacy from Ca supplementation (not dietary sources of Ca) is inconsistent, likely due to the same limitations observed in dairy food-based studies; participants not being Ca deficient, poor compliance, high dropout rates and insufficient number of participants and study duration for enough fracture events to occur for efficacy to be detectable^(^ ^33^ ^)^. Sub-analyses of these trials are suggestive of anti-fracture efficacy. When the effect of Ca supplementation was tested in subgroups with intake <800 mg/d, a 20 % reduction in fracture risk was observed and reduced fracture rates have been observed in *post hoc* analyses in those with low Ca intake in the community and in aged-care^(^ ^69^ ^–^ ^71^ ^)^.

To demonstrate the anti-fracture efficacy of dairy food consumption, the same stringent design criteria highlighted earlier needs to be applied: large sample size, low baseline Ca/dairy food intakes, good compliance and limited dropouts. Such a trial in the community would be difficult to conduct and likely not feasible as the numbers needed to treat to prevent a fracture event would be large, compliance difficult to monitor and verifying low-trauma fractures problematic as self-reporting and recall of fracture events are often unreliable especially in those with cognitive impairment^(^ ^72^ ^)^. The setting in which the successful execution of a dairy food intervention is most probable is in institutionalised elderly. In this group, fracture risk is the highest compared with any other group in the community, nutritional deficiencies prevail, food intake (and compliance) can be closely monitored and fractures recorded as the setting in well controlled^(^ ^73^ ^,^ ^74^ ^)^. The success of the landmark Ca and vitamin D fracture trial was likely driven by the low Ca intake and high levels of vitamin D deficiency in participants^(^ ^75^ ^)^.

Dairy food-based interventions conducted in this setting to date are promising (Table 1). Bonjour *et al.* ^(^ ^76^ ^)^ compared the efficacy of vitamin D- (10 µg) and Ca-fortified (800 mg) yogurt to regular yogurt (Ca content 280 mg) over 56 d in sixty vitamin D-deficient elderly institutionalised women and observed parathyroid hormone and CTX were 20 % (*P*<0·001) and 8 % (*P*<0·05) lower, respectively, with fortification. A 42 % (OR=0·58, 95 % CI 0·44, 0·78, *P*<0·001) reduction in the number of institutionalised elderly who fell was observed after 12 months of supplementation using a dairy food-based protein, Ca and vitamin D supplement in 813 aged-care residents^(^ ^77^ ^)^. The study was not powered to detect fractures. Using a dairy food-based approach in the aged-care setting, dairy food intake was increased from two to four servings daily, resulting in a doubling of Ca intake (679 mg, *P*<0·0001), the consumption of 25 g/d more protein daily (*P*<0·0001) and residents meeting protein and Ca requirements, which remained below the recommended intake levels in controls^(^ ^78^ ^)^.

The existing evidence from dairy food intervention trials is suggestive of an anti-fracture efficacy of enhanced dairy food consumption. Given the widespread availability of dairy foods, it is a reasonable and potentially feasible public health approach to fracture prevention. Modelling of the economic impact of dairy food supplementation to reduce fractures indicates that it is cost-effective in postmenopausal women aged over 70 years, particularly in those with low Ca intake (<600 mg/d)^(^ ^79^ ^)^. These public health approaches need concerted consideration as the population ages and the proportion of high-risk older adults increases. However, until a well-designed dairy food intervention trial is conducted, the anti-fracture benefits of dairy food consumption will continue to be based on conjecture.

## Acknowledgements

Funding was provided by the Dairy Council (www.milk.co.uk). The Dairy Council GB had no role in the writing of this manuscript.

S. I. and T. R. H. were responsible for the preparation and writing of this review.

S. I. receives research funding support from Dairy Australia, California Dairy Research Foundation, National Dairy Council, Aarhus University Hospital and Danish Dairy Research Foundation, Fonterra Co-operative Group Ltd, Dutch Dairy Association, Dairy Council of California, Dairy Farmers of Canada and the Centre National Interprofessionnel de l’Economie Laitiere. T. R. H. has received consultancy fees and honoraria from the Dairy Council GB.
